# Single Sodium Pyruvate Ingestion Modifies Blood Acid-Base Status and Post-Exercise Lactate Concentration in Humans

**DOI:** 10.3390/nu6051981

**Published:** 2014-05-16

**Authors:** Robert A. Olek, Sylwester Kujach, Damian Wnuk, Radoslaw Laskowski

**Affiliations:** 1 Biochemistry Department, Gdansk University of Physical Education and Sport, Gorskiego 1, 80-336 Gdansk, Poland; 2 Physiology Department, Gdansk University of Physical Education and Sport, Gorskiego 1, 80-336 Gdansk, Poland; E-Mails: sylwek-kujach@o2.pl (S.K.); lasradek@awf.gda.pl (R.L.); 3 Department of Physiotherapy, Medical University of Gdansk, Debinki 7, 80-211 Gdansk, Poland; E-Mail: damianwnuk@gumed.edu.pl

**Keywords:** sodium pyruvate, exercise, blood acid-base status, lactate, alanine

## Abstract

This study examined the effect of a single sodium pyruvate ingestion on a blood acid-base status and exercise metabolism markers. Nine active, but non-specifically trained, male subjects participated in the double-blind, placebo-controlled, crossover study. One hour prior to the exercise, subjects ingested either 0.1 g·kg^−1^ of body mass of a sodium pyruvate or placebo. The capillary blood samples were obtained at rest, 60 min after ingestion, and then three and 15 min after completing the workout protocol to analyze acid-base status and lactate, pyruvate, alanine, glucose concentrations. The pulmonary gas exchange, minute ventilation and the heart rate were measured during the exercise at a constant power output, corresponding to ~90% 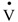 O_2_max. The blood pH, bicarbonate and the base excess were significantly higher after sodium pyruvate ingestion than in the placebo trial. The blood lactate concentration was not different after the ingestion, but the post-exercise was significantly higher in the pyruvate trial (12.9 ± 0.9 mM) than in the placebo trial (10.6 ± 0.3 mM, *p* < 0.05) and remained elevated (nonsignificant) after 15 min of recovery. The blood pyruvate, alanine and glucose concentrations, as well as the overall pulmonary gas exchange during the exercise were not affected by the pyruvate ingestion. In conclusion, the sodium pyruvate ingestion one hour before workout modified the blood acid-base status and the lactate production during the exercise.

## 1. Introduction

Pyruvate (PYR) participates in several various reactions and plays a key role in energy metabolism. It is produced from glucose (GLU) in the cytoplasm of skeletal muscle cells by the glycolytic pathway. During this process, NAD^+^ is reduced to NADH in the reaction catalyzed by glyceraldehyde-3-phosphate dehydrogenase (G3PDH). The slow rate of glycolysis in the low intensity exercise allows all produced PYR and reducing equivalents to be transferred to mitochondria. Inside the mitochondria, PYR is decarboxylated by the pyruvate dehydrogenase complex (PDC) to acetyl-CoA, which enters the tricarboxylic acid (TCA) cycle. PDC and TCA cycle dehydrogenases generate reducing equivalents used for ATP production in the oxidative phosphorylation process performed in the electron transport chain. On the other hand, a high energy demand during an intensive exercise raises the rate of PYR production. PYR is reduced to lactate (LA) by the cytoplasmic NADH in the reaction catalyzed by the lactate dehydrogenase (LDH). This mechanism enables PYR to increase the cytosolic ATP resynthesis rate (for a review, see [1]). The exercise intensity elevation also causes a higher release of alanine (ALA) from the skeletal muscles [2]. ALA is produced by PYR transamination. In the same reaction, α-ketoglutarate, an intermediate of TCA cycle, is formed.

It has been shown that PYR diminishes cellular and organ dysfunction during hypoxic conditions [3,4,5,6,7]. The increased LA production [4] and the direct NADH measurement [3] indicate that the presence of exogenous PYR improves the cytosolic NADH reoxidation. Therefore, the blockage of G3PDH by a high NADH/NAD^+^ ratio is moderated, and ATP production by anaerobic glycolysis may be stimulated [8]. Moreover, Robergs *et al.* [9] suggested that PYR reduction to LA consumes H^+^; therefore, PYR may act as a buffer. The buffering properties of PYR have been directly shown, while it has been used in a perfusing solution of the isolated organs [4,8] or during intravenous infusion [5,6,7,10]. However, in all these studies, PYR has been used in supraphysiological concentrations [4,5,6,7,8,10].

Despite the importance of PYR in energy metabolism, no ergogenic effects have been shown after a prolonged supplementation [11,12]. PYR intake for seven days failed to enhance the performance during an intense aerobic exercise in well-trained individuals [11]. Furthermore, PYR consumption for 14 consecutive days does not improve exercise capacity, as measured by a critical power cycle ergometer test [12]. Moreover, Morrison *et al.* [11] indicated that acute oral intake of calcium PYR (CaP), even at a dose of 25 g, did not modify the PYR concentration in the whole blood or in the plasma. However, we have recently found that single sodium PYR (NaP) and CaP ingestion increased blood pH, but NaP was more effective in the blood bicarbonate level modification [13] (unpublished). Therefore, the purpose of this study is to examine the effect of a single NaP ingestion on blood acid-base status and the exercise metabolism markers.

Since 0.1 g of sodium bicarbonate per kg of body mass induces metabolic alkalosis 60 min following ingestion [14,15], we hypothesized that a similar NaP treatment before commencing the high intensity physical exertion may change the exercise metabolism.

## 2. Experimental Section

### 2.1. Subjects

Nine active, but non-specifically trained, male subjects (mean ± SEM: 23 ± 1 year old, 1.75 ± 0.02 m height, 72 ± 2 kg body mass) participated in the double-blind, placebo-controlled, crossover study. The study was approved by the Local Ethics Committee, and all subjects signed an informed consent before the start of the study. The subjects were asked to refrain from any physical activity or alcohol consumption for at least 24 h and caffeine for at least 12 h prior to testing.

### 2.2. Procedures

To determine 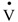 O_2_max, participants performed a graded cycle ergometry test on an electromagnetically-braked, cycle ergometer (ER 900 Jaeger, Viasys Healthcare GmbH, Hoechberg, Germany). The height of the ergometer seat was individually adjusted, and the participants were allowed a 5-min warm-up period at an intensity of 1.5 W·kg^−1^ with a pedaling cadence of 60 rpm. After the warm-up period, the work rate was increased by 25 W·min^−1^ until volitional exhaustion [16]. Breath by breath pulmonary gas exchange was measured by Oxycon-Pro analyzer (Viasys Healthcare GmbH, Hoechberg, Germany), and the O_2_ and CO_2_ analyzers were calibrated prior to each test using standard gases of known concentrations in accordance with manufacturer guidelines. The heart rates were monitored continuously by telemetry (S-625, Polar Electro-Oy, Kempele, Finland) during each test session and the first 5 min of passive recovery in a seated position. After the 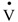 O_2_max test, subjects visited the laboratory for a practice ride, which familiarized the subjects with the experiment protocol and confirmed the power output (~90% 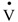 O_2_max).

### 2.3. Measurements

On separate days, the subjects reported to the laboratory in the morning, then rested for 30 min and then ingested placebo or NaP in a random order. In the previous studies the subjects consumed PYR in the amount of ~0.07–0.08 g·kg^−1^ body mass [17,18]; therefore, the subjects in our study ingested NaP in a single dose of 0.1 g·kg^−1^ body mass (which is ~0.08 g of PYR per kg body mass). Moreover, it has been reported that the same dose of sodium bicarbonate induces metabolic alkalosis [14,15]. An hour following the ingestion, the subjects performed the physical exertion. The exercise protocol consisted of 2 min at a power output of 50 W and then for 6 min at a constant power output, corresponding to ~90% 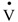 O_2_max. The respiratory gas analysis and the volume measurements were performed breath by breath with a face-mask connected to the analyzer. The breath-by-breath pulmonary 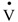 O_2_ was measured continuously throughout the exercise by an Oxycon-Pro gas analyzing system (Viasys Healthcare GmbH, Hoechberg, Germany). The data were first manually filtered to remove outlying breaths, defined as breaths deviating by more than three standard deviations from the preceding five breaths. The data were subsequently interpolated to provide second-by-second values, and then, the slow component amplitude was estimated by calculating the difference between the mean 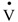 O_2_ during the last 60 s of the exercise and the mean 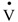 O_2_ during the 60-s period on third minute of exercise [19].

### 2.4. Blood Analysis

At rest, 60 min after NaP or the placebo ingestion, and then 3 and 15 min after completing the exercise protocol, the blood samples were taken from the fingertips into heparinized capillary tubes (Siemens Healthcare Diagnostics Inc., Tarrytown, NY, USA) and were immediately analyzed for blood gases, pH, hemoglobin (Hb) and electrolytes using a Rapidpoint 400/405 (Siemens Healthcare Diagnostics Inc., Tarrytown, NY, USA). In addition, the bicarbonate (HCO_3_^−^) was calculated from PCO_2_ and pH values according to the Henderson–Hasselbalch equation, and the base excess (BE) was calculated according to the following equation [20]:

BE = (1 − 0.014 × [Hb]) × ([HCO_3_^−^] − 24.8 + (1.43 × [Hb] + 7.7) × (pH − 7.4)) (1)

The blood samples for GLU, LA, PYR and ALA analysis were immediately deproteinized by the addition of ice cold 0.4 M perchloric acid. After being thoroughly mixed, the samples were centrifuged at 12,000× *g* for 10 min and stored for later analysis. GLU concentration was quantified using a standard test kit (Randox Laboratories Ltd., Crumlin, UK) based on the glucose oxidase method (GL2623). Assays were performed on Super Aquarius CE9200 (Cecil Instruments Ltd., Cambridge, UK). LA, PYR and ALA concentrations were determined fluorometrically by the method of Maughan [21], which is based on the enzymatic reactions of NAD^+^ reduction (LA, ALA) or NADH oxidation (PYR). The fluorescence intensity was measured at an excitation maximum of 340 nm and an emission maximum of 460 nm on an EnSpire Multimode Plate Reader (Perkin Elmer Inc., Waltham, MA, USA). The known concentrations of the compounds were used to construct a standard curve. All the reagents were obtained from Sigma-Aldrich Co. (Schnelldorf, Germany).

### 2.5. Statistics

The statistical analyses were performed using STATISTICA 9.0 (Statsoft Inc., Tulsa, OK, USA) software, with the level of significance set at *p* < 0.05. A repeated-measures ANOVA across treatments (placebo and NaP) and time (rest, 60 min after ingestion, 3 min after the exercise and 15 min of recovery) were used to assess the differences in the blood acid-base status and metabolites. Furthermore, due to the small number of subjects, we also calculated the effect size (partial η^2^). It is a measure of effect size for use in ANOVA, ranging between 0 and 1. Using Cohen’s rule of thumb, as well as the conversion table for η^2^, the interpretations of the partial η^2^ value are unequivocal. However, the most restricted interpretation method assigns values to the effect size as follows: 0.1, a small effect; 0.3, a medium effect; and 0.5, a large effect. The pulmonary gas exchange parameters were analyzed applying a Student’s *t*-test.

## 3. Results

**Figure 1:**
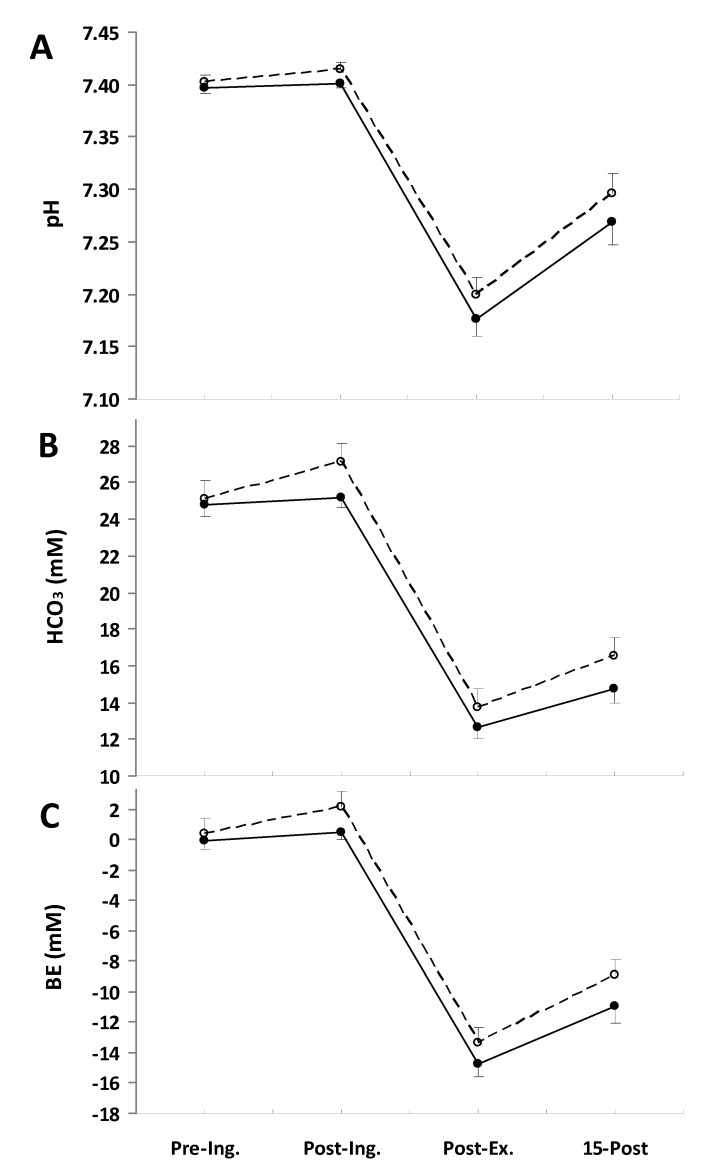
Blood pH (**A**), HCO_3_ (**B**), and base excess (BE) (**C**) responses measured before placebo (●) and NaP (○) ingestion (Pre-Ing.), 60 min after ingestion (Post-Ing.), 3 min after completion of exercise (Post-Ex.) and 15 min of recovery (15-Post). Values are the means ± SEM.

The ingestion of NaP affected the blood acid-base status (Figure 1). The pH was significantly higher in the NaP trial than in the placebo trial (*p* = 0.015, partial η^2^ = 0.54; Figure 1A). Similarly, the blood bicarbonate concentration (Figure 1B) and BE (Figure 1C) increased after NaP ingestion and remained above the placebo conditions. The main treatment effects for HCO_3_^−^ and BE were (*p* = 0.005, partial η^2^ = 0.65; and *p* = 0.003, partial η^2^ = 0.69), respectively.

**Figure 2:**
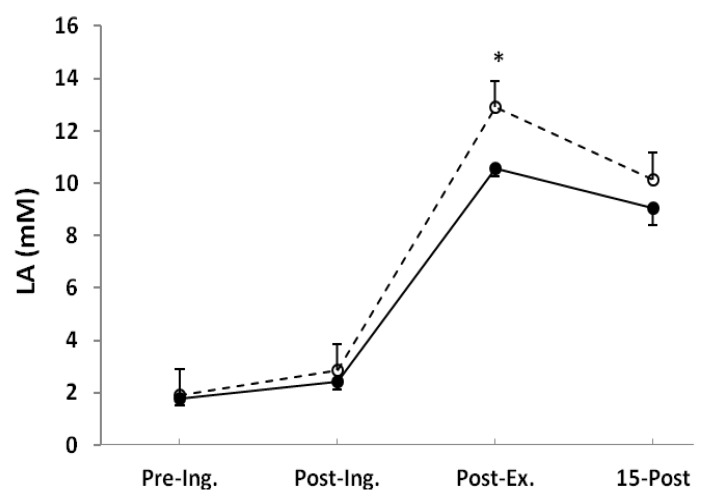
Blood lactate (LA) measured before placebo (●) and NaP (○) ingestion (Pre-Ing.), 60 min after ingestion (Post-Ing.), 3 min after completion of exercise (Post-Ex.), and 15 min of recovery (15-Post). Values are the means ± SEM. * Significantly different from placebo at the same time point (*p* < 0.05).

Blood LA concentration was not different after ingestion, but post-exercise was significantly higher in the NaP (12.9 ± 0.9 mM) than in the placebo (10.6 ± 0.3 mM, *p* < 0.05) and remained elevated (nonsignificant) after 15 min of recovery (Figure 2).

The main effect of time (*p* < 0.001) was observed in PYR, ALA and GLU concentrations in the blood (Table 1). However, there were no significant differences between the trials; NaP ingestion had no effect on measured metabolites (Table 1).

**Table 1:** Blood pyruvate (PYR), alanine (ALA) and glucose (GLU) concentrations at rest, after placebo or NaP ingestion and following exercise. Values are the means ± SEM.

	Placebo	NaP
PYR (μM) ^#^		
Rest	101 ± 18	110 ± 33
60 min after ingestion	149 ± 22	191 ± 51
3 min after exercise	329 ± 34	344 ± 34
15 min after exercise	354 ± 42	300 ± 69
ALA (μM) ^#^		
Rest	296 ± 21	286 ± 28
60 min after ingestion	285 ± 24	331 ± 23
3 min after exercise	375 ± 53	385 ± 34
15 min after exercise	383 ± 21	427 ± 45
GLU (mM) ^#^		
Rest	52.0 ± 1.4	51.7 ± 1.4
60 min after ingestion	51.8 ± 0.8	51.7 ± 1.5
3 min after exercise	63.3 ± 2.0	63.3 ± 2.8
15 min after exercise	58.8 ± 1.4	57.2 ± 2.9

^#^ Main effect of time (*p* < 0.001).

The NaP ingestion resulted in no alterations in the overall 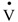 O_2_ response during the exercise (Table 2). Consistent with this, the slow component amplitude was not significantly different between treatments. There were also no marked differences in 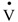 CO_2_ during the final minute of the exercise, being 4.08 ± 0.10 L·min^−1^ in the placebo trial and 4.03 ± 0.09 L·min^−1^ in the NaP trial; therefore, the respiratory exchange ratio (RER) was also not altered by the NaP ingestion (Table 2). Furthermore, no effects of NaP were noted in 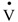 E (Table 2).

**Table 2:** Gas exchange, ventilation and heart rate responses during and after severe-intensity exercise following placebo and sodium pyruvate ingestion. Values are the means ± SEM.

	Placebo	NaP
O_2_ uptake, L min^−1^		
Baseline	1.07 ± 0.02	1.01 ± 0.05
End-exercise	3.52 ± 0.06	3.44 ± 0.06
Slow component amplitude	0.53 ± 0.04	0.50 ± 0.04
CO_2_ output, L min^−1^		
Baseline	0.81 ± 0.03	0.78 ± 0.04
End-exercise	4.08 ± 0.10	4.03 ± 0.09
Minute ventilation, L min^−1^		
Baseline	23 ± 1	22 ± 1
End-exercise	121 ± 5	116 ± 6
Respiratory exchange ratio		
Baseline	0.76 ± 0.02	0.77 ± 0.02
End-exercise	1.16 ± 0.02	1.17 ± 0.02
Heart rate, beats min^−1^		
Baseline	86 ± 3	87 ± 4
End-exercise	171 ± 2	171 ± 3

## 4. Discussion

The major new finding of this study was that the single oral NaP ingestion modified the blood acid-base status. Moreover, the post-exercise blood LA concentration was higher in the NaP trial. The gas exchange and the ventilation responses were not affected by the NaP consumption.

The buffering character of PYR has been presented in earlier studies [5,6,7,22]. However, previously, PYR was infused intravenously at very high doses [5,6,7,22]. The intravenous infusion raised the arterial PYR concentration from 0.12 to 5.68 mM 30 min after the start of the infusion. At the same time, blood pH, BE and HCO_3_^−^ reached 7.54, 12 mM and 38 mM, respectively [5]. In the present study, we noted the modification in the blood acid-base status, but without any alteration in the blood PYR concentration. Similarly, Morrison *et al.* [11] showed that an acute oral PYR intake, even at a dose of 25 g, did not modify PYR concentration in the whole blood or in the plasma. The inability to detect any elevation of PYR in blood, as well as the increased borborygmus and flatulence in the subjects consuming higher doses led Morrison and coworkers to suggestion that it could be decarboxylated in the stomach or eliminated through the feces; however, rapid clearance by the liver or muscles has not been ruled out [11]. Since the blood acid-base status has been ameliorated after an oral NaP ingestion, its absorption from the digestive system may occur.

It has been postulated that the buffering PYR mechanism may be based on its transport to the cells [23]. The uptake of PYR by the cells depends on the monocarboxylate transporter system. This system is located in the plasma membrane, and it transports monocarboxylates together with H^+^ [24]. The transfer of the hydrogen proton into the cytosol effectively raises the blood pH. Since the intravenous PYR infusion failed to increase muscle PYR content [25], it would be expected that the majority of PYR would be cleared by the liver [11]. Considering PYR as an excellent gluconeogenic precursor, it can be either released as GLU or stored as glycogen. Although there is no significant differences in blood GLU over time after oral intake [11] or intravenous infusion [25], the trend toward the higher RER values during a low intensity exercise, suggesting a higher rate of carbohydrate oxidation after PYR supplementation, has been presented [18].

It has been also proposed that the PYR buffering properties derived from its ability to be reduced to LA. During this reaction, H^+^ is utilized [9,23]. However, the stable blood LA over 4 h rest, even after ingestion of 25 g PYR, has been previously observed [11]. Since after a high intensity exercise, the cytoplasmic NADH/NAD^+^ ratio in the skeletal muscle elevates [26], it seems that PYR could react with the excess of NADH, forming LA. The increased LA level in the NaP trial may indicate that ingested PYR could be partially reduced to LA. However, despite an increased LA concentration, the LA/PYR ratio was not different between the trials (not shown). Moreover, the effect of NaP was similar to that previously reported after sodium bicarbonate treatment; the modification of blood acid-base status and the elevation of a post-exercise LA concentration [27,28,29,30].

The results of the studies examining the net exchange of PYR across working skeletal muscle in humans reported that the muscles release PYR both during long exercise at lower intensities [31,32] and a short duration, high intensity workout [33]. In the present study, the arterio-venous difference was not determined, but capillary blood PYR concentration increased after exercise. However, no difference between the trials was noted.

Another metabolic fate of PYR in a working skeletal muscle is the conversion to ALA [2]. In the reaction catalyzed by the glutamate-pyruvate transaminase (GPT), the amino group is transferred from glutamate to PYR, resulting in ALA and α-ketoglutarate formation. During strenuous exercise, the production of ALA by muscles markedly increases [34,35]. However, daily 25 g of NaP in a combination with 75 g of dihydroxyacetone supplementation for a week do not affect the post-exercise blood ALA concentration [36]. Similarly, in the present study, the blood ALA concentration was not affected by the NaP ingestion.

The GPT reaction in a skeletal muscle is not only essential for ALA synthesis. Due to the production of α-ketoglutarate, it may play an important role in replenishing the TCA intermediates in the muscle mitochondria at the onset of exercise [37]. The findings of Timmons *et al.* [38] suggest that the provision of the oxidative substrate is a factor that limits oxidative metabolism early in exercise and that increasing the availability of the substrate early in exercise allows for increased oxidative metabolism. However, the later studies indicated that a modification in TCA intermediate concentration does not affect the oxygen consumption [39,40] nor the mitochondrial respiration [41].

The slow component of the oxygen uptake kinetics represents an increasing oxygen (and energy) cost during exercise, despite the rate of the external work remaining constant, and may be implicated in the fatigue process [42]. The interventions that reduce the 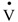 O_2_ slow component amplitude have been reported to improve the tolerance of severe intensity exercise [43,44]. However, the results of the pre-exercise alkalinization by oral sodium bicarbonate ingestion are equivocal. Some authors found a significant reduction of the slow component [28,45], whereas others observed no effect [46,47]. In the present study, NaP did not influence the slow component amplitude.

The PYR supplementation has no ergogenic effect after seven or 14 days [11,12]. The PYR doses used in these studies are about ~0.1 g·kg^−1^·day^−1^ [11,12]. Moreover, this amount has been divided into four aliquots; 2 g, three times a day and 1 g with a small snack before going to bed [11]. On the contrary, high dosages of PYR in combination with dihydroxyacetone (25 g and 75 g per day, respectively) consumed for one week enhance the endurance capacity [48,49], but such a supplementation protocol may be distressful to the stomach.

## 5. Conclusions

Although the results showed an improvement of the blood acid-base parameters after a single dose of NaP ingestion, it should be noted that the limitation of the present study was the lack of its effect on performance. However, it seems plausible that single PYR ingestion could have an ergogenic capacity, especially ingested prior to high-intensity physical efforts. Therefore, further experimental work would be necessary to clarify the mechanism of PYR action.
